# Cognitive complaints mediate childhood parental bonding influence on presenteeism

**DOI:** 10.1371/journal.pone.0266226

**Published:** 2022-03-29

**Authors:** Kuniyoshi Toyoshima, Takeshi Inoue, Akiyoshi Shimura, Jiro Masuya, Yota Fujimura, Shinji Higashi, Ichiro Kusumi

**Affiliations:** 1 Department of Psychiatry, Hokkaido University Graduate School of Medicine, Sapporo, Japan; 2 Department of Psychiatry, Tokyo Medical University, Shinjuku-ku, Tokyo, Japan; 3 Department of Psychiatry, Tokyo Medical University Hachioji Medical Center, Tokyo, Japan; 4 Department of Psychiatry, Ibaraki Medical Center, Tokyo Medical University, Ami-machi, Inashiki-gun, Ibaraki, Japan; Osaka City University: Osaka Shiritsu Daigaku, JAPAN

## Abstract

**Background:**

Childhood parental bonding and cognitive complaints (CCs) affect a worker’s mental health (MH), and CCs affect presenteeism. However, the impact of childhood parental bonding on presenteeism and the mediating effect of CCs with respect to the association among childhood parental bonding and presenteeism remain poorly understood.

**Aim:**

We aimed to investigate the mediating role of CCs on the relationship between childhood parental bonding and presenteeism to better understand the influence of childhood parental bonding on adulthood presenteeism.

**Setting:**

A total of 440 Japanese adult workers recruited using convenience sampling were evaluated.

**Methods:**

The Parental Bonding Instrument, Cognitive Complaints in Bipolar Disorder Rating Assessment and Work Limitations Questionnaire 8 were used to assess childhood parental bonding, CCs, and presenteeism, respectively. We performed Spearman’s correlation analysis and path analysis to investigate the relationship among the variables.

**Results:**

Path analysis revealed that childhood parental bonding and CCs significantly affected presenteeism. More specifically, CCs fully and partially mediated the effect of paternal and maternal care on presenteeism, respectively. Moreover, CCs partially mediated the effects of both paternal and maternal overprotection on presenteeism.

**Conclusion:**

The mediating role of CCs on the relationship between childhood parental bonding and presenteeism was shown in this study. In occupational MH, evaluating the mediating effect of CCs may be useful for addressing adulthood presenteeism associated with childhood parental bonding.

## Introduction

The importance of workplace mental health (MH) has increased in recent years [1]. The pandemic has affected the MH of healthcare workers, causing depression, insomnia and anxiety [2]. A recent meta-analysis reported a high prevalence of depressive symptoms (DSs) among nurses [3]. Regarding interventions, a systematic review suggested the effectiveness of compassion fatigue interventions in the MH of workers [4]. Furthermore, a pilot randomized controlled trial showed that work-directed rehabilitation improved the work ability and MH of individuals with mental illness [5]. However, effective interventions for the MH of workers have not yet been established at this stage.

Childhood parental bonding influences MH in adulthood. In the general adult population, low care and high overprotection affect DSs indirectly via neuroticism [6]. Furthermore, poor quality parenting indirectly exacerbates DSs via subjective social status and self-esteem [7]. Hence, prevention and management of adverse childhood experiences are required in public health [8]. Regarding the influence of childhood parental bonding on the MH of workers, childhood parental bonding affects the stress-coping ability and reactions to stress of adult workers [9]. However, the relationship between childhood parental bonding and presenteeism (i.e. going to work despite feeling unhealthy and the health-related loss of work productivity) remains poorly understood.

Cognitive disturbance is a serious concern in occupational MH, because it can lead to loss of work productivity [10]. Cognitive function affects various situations in daily life, and cognitive disturbance is often noticed by the individuals themselves [11]. Cognitive complaints (CCs) mean subjective cognitive disturbance, and CCs may also be described as subjective cognitive dysfunction [12]. Objective cognitive disturbance is the opposite of subjective cognitive disturbance, which can be evaluated, for example, by neuropsychological tests [13]. Previous studies have shown that subjective and objective cognitive disturbance do not always match, often diverging in individuals with psychiatric illness [14, 15]. Although, it is necessary to consider this fact when subjectively assessing cognitive disturbance, evaluation of CCs is useful in public health because it can be easily performed within a short period of time compared with the evaluation of objective cognitive disturbance [11, 16]. Moreover, CCs are more closely associated with quality of life than objective cognitive dysfunction [17, 18]. Hence, recently, the importance of CCs in public and occupational MH has increased.

CCs are associated with various clinical factors. In adults, DSs, affective temperaments and childhood parental bonding are important factors influencing CCs [19]. Moreover, CCs affect social function, life quality, and presenteeism [10, 11]. Recently, the mediating effects of CCs were reported. In adults, CCs mediate the impact of DSs on life quality [11]. Further, CCs mediate the impact of temperament characteristics on social functioning [20]. In workers, CCs mediate the impact of DSs on presenteeism [10]. Hence, the mediating roles of CCs have been evaluated in public and occupational MH. However, to our knowledge, the mediating role of CCs on the relationship between childhood parental bonding and presenteeism remains unclear. We hypothesized that CCs mediate the influence of childhood parental bonding on presenteeism, and the mediating effect of CCs may differ depending on the type of parental bonding. Therefore, in Japanese adult workers, we aimed to verify the hypothesis using path analysis.

## Materials and methods

### Participants

A total of 597 adult volunteers were conveniently recruited between April 2017 and April 2018 in Tokyo, Japan. Our study was part of a larger study, where several self-administered scales were investigated [11]. Approval by the Local Ethics Committee of Tokyo Medical University (Ethics Approval Number: SH3502) was obtained in advance. This research was performed in accordance with the Declaration of Helsinki as revised in 1989. This paper does not disclose any personal identifiable information of any of the participants in any form. Hence, consent for publication is not applicable here. Written informed consent for participation was provided by all participants. The final sample consisted of 440 workers, because 157 participants did not complete the assessments or were unemployed.

#### Self-administered scales

*CCs*. The Cognitive Complaints in Bipolar Disorder Rating Assessment (COBRA) consists of 16 items, which can evaluate CCs [12]. Each item can be evaluated from 0 (never) to 3 (always). The total score was determined by adding each score. High scores indicate severe CCs. This study used the Japanese version, which was validated and used in adults [11, 13].

*Parental bonding*. The Parental Bonding Instrument (PBI) composed of 25 items, which evaluate parenting styles [21]. It contains the four sub-scores: maternal care (MCA), paternal care (PCA), maternal overprotection (MOP), and paternal overprotection (POP). The high score shows the higher level of perceived parenting style (e.g. high PCA score indicates great care from the father). The PBI was shown to be stable over 20 years [22]. We used the validated Japanese version [23].

#### Presenteeism

The Work Limitations Questionnaire 8 (WLQ-8) consists of eight items, which can evaluate working disability; it is a short version of the Work Limitations Questionnaire 25 [24, 25]. The Work Limitations Questionnaire (WLQ)-8 Japanese version was used to analyse presenteeism by calculating the work productivity loss score to estimate the percentage of presenteeism [10, 26]. High work productivity loss scores indicate severe presenteeism.

### Statistical analysis

Spearman’s correlation analysis using Bonferroni correction was performed to investigate the associations among parental bonding, CCs, and presenteeism. The path analysis was performed to investigate the mediating effect of CCs on the relationship among parental bonding and presenteeism. This study did not demonstrate the goodness-of-fit index because of a saturation model. The standardized path coefficients were analyzed to demonstrate the strengths of the effects. We performed additional path analysis after omitting data of the participants with current psychiatric treatment and psychiatric history (non-psychiatric group; N = 390). All statistical analyses were conducted with the IBM SPSS Statistics for Windows, Version 21.0 (IBM Corp, Armonk, NY, USA) and STATA/MP 16 (StataCorp LLC, College Station, TX, USA) software; *p* < 0.05 denoted statistically significant differences.

## Results

### Sociodemographic characteristics

The basic findings are demonstrated in Table 1. At the time of the investigation, all participants were employed. In this study, the quality of parental bonding was evaluated by PBI, CCs were evaluated by the COBRA total score, and presenteeism was evaluated by the work productivity loss score.

**Table 1:** Sociodemographic characteristics (N = 440).

Characteristic	Mean (Standard deviation)	Number (%)
Age	40.8 (11.7)	
Gender (female)		245 (55.7)
Marital status (yes)		283 (64.3)
Education (years)	14.8 (1.8)	
Psychiatric history (yes)		46 (10.5)
Current psychiatric treatment (yes)		19 (4.3)
Alcoholic (yes)		291 (66.1)
Smoking (yes)		83 (18.9)
PBI sub-scores		
Paternal care	23.8 (7.9)	
Paternal overprotection	9.6 (6.7)	
Maternal care	28.3 (6.8)	
Maternal overprotection	9.6 (6.9)	
CCs (COBRA total)	8.4 (6.5)	
Presenteeism (WLQ work productivity loss)	0.04 (0.04)	

CCs, cognitive complaints; COBRA, Cognitive Complaints in Bipolar Disorder Rating Assessment; PBI, Parental Bonding Instrument; SD, standard deviation; WLQ, Work Limitations Questionnaire.

### Spearman’s correlation analysis

The results of the Spearman’s correlation analyses are demonstrated in Table 2. PCA significantly and negatively correlated with CCs, whereas PCA did not significantly correlate with presenteeism (*p* = 0.072). MCA significantly and negatively correlated with CCs and presenteeism. However, POP and MOP significantly and positively correlated with CCs and presenteeism.

**Table 2:** Spearman’s correlation analysis (*rho*) (N = 440).

	PCA	POP	MCA	MOP	CCs	Presenteeism
PCA	-					
POP	−0.57***	-				
MCA	0.55***	−0.50***	-			
MOP	−0.41***	0.67***	−0.60***	-		
CCs	−0.20***	0.24***	−0.20***	0.25***	-	
Presenteeism	−0.13	0.23***	−0.16**	0.21***	0.46***	-

CCs, cognitive complaints; MCA, maternal care; MOP, maternal overprotection; PCA, paternal care; POP, paternal overprotection.

*p < 0.05

**p < 0.01

***p < 0.001 (two-sided).

### Path analysis

**Fig 1:**
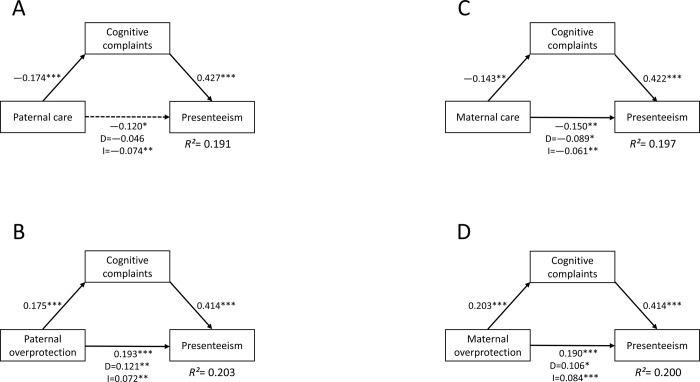
The mediating effect of cognitive complaints on the relationship between childhood parental bonding and presenteeism in adult workers (N = 440). D, direct effect; I, indirect effect; *R*^*2*^, coefficient of determination. Relationships between childhood parental bonding, CCs, and presenteeism are demonstrated using path analyses. A) CCs fully mediated the impact of paternal care on presenteeism. B) CCs partially mediated the impact of paternal overprotection on presenteeism. C) CCs partially mediated the impact of maternal care on presenteeism. D) CCs partially mediated the impact of maternal overprotection on presenteeism. The numbers beside the arrows demonstrate the standardized path coefficients of total effects. The numbers below the total effect of parental bonding on presenteeism demonstrate the direct (D) and indirect (I) effects. The solid line demonstrates a significant direct effect, and the dotted line demonstrates a non-significant direct effect. Parental bonding demonstrates PBI sub-scores; CCs demonstrate COBRA total score; and presenteeism demonstrates WLQ work productivity loss score.

The results of the path analyses for a total sample (N = 440) are demonstrated in Fig 1. PCA significantly and negatively influenced presenteeism indirectly via CCs. POP and MOP significantly and positively influenced presenteeism directly and indirectly via CCs. MCA significantly and negatively influenced presenteeism directly and indirectly via CCs. In summary, CCs fully mediated the influence of PCA on presenteeism and partially mediated the influence of POP, MCA, and MOP on presenteeism.

**Fig 2:**
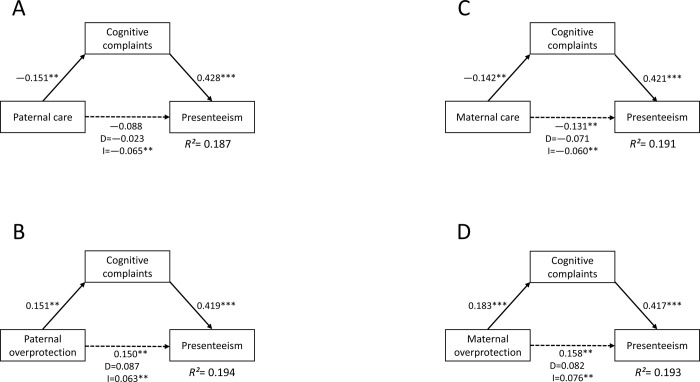
The mediating effect of cognitive complaints on the relationship between childhood parental bonding and presenteeism in non-psychiatric adult workers (N = 390). D, direct effect; I, indirect effect; *R*^*2*^, coefficient of determination. Relationships between childhood parental bonding, CCs, and presenteeism are demonstrated using path analyses. A) Paternal care directly affected CCs, and CCs directly affected presenteeism. B) CCs fully mediated the impact of paternal overprotection on presenteeism. C) CCs fully mediated the impact of maternal care on presenteeism. D) CCs fully mediated the impact of maternal overprotection on presenteeism. The numbers beside the arrows demonstrate the standardized path coefficients of total effects. The numbers below the total effect of parental bonding on presenteeism demonstrate the direct (D) and indirect (I) effects. The solid line demonstrates a significant direct effect, and the dotted line demonstrates a non-significant direct effect. Parental bonding demonstrates PBI sub-scores; CCs demonstrate COBRA total score; and presenteeism demonstrates WLQ work productivity loss score.

The results of the additional path analyses for non-psychiatric adult workers (N = 390) are demonstrated in Fig 2. PCA, POP, MCA, and MOP did not significantly influence presenteeism directly, whereas all of them significantly influenced presenteeism indirectly via CCs. The total effects of POP, MCA, and MOP on presenteeism were statistically significant, while that of PCA was not.

## Discussion

The findings of this study suggest that CCs mediate the effect of parental bonding on presenteeism. In terms of the path analysis, “CCs→presenteeism” was already reported in our previous study [10]. However, “PCA, POP, MCA, MOP→CCs” and “PCA, POP, MCA, MOP→presenteeism” were first reported in the present study. In other words, the common path was only “CCs→presenteeism,” and other eight paths were newly investigated in this study. To our knowledge, our study firstly suggests the mediating role of CCs on the relationship among parental bonding and presenteeism in adult workers. A previous study suggested that DSs exacerbate the CCs and presenteeism, and CCs partially mediate the effect of DSs on presenteeism [10]. In the present study, POP and MOP exacerbated CCs and presenteeism, and CCs partially mediated the effects of POP and MOP on presenteeism. Hence, regarding the mediating role of CCs on presenteeism, the influence of DSs may be similar to those of POP and MOP. In the general population, parental overprotection (i.e. POP and MOP) exacerbates DSs indirectly via neuroticism [6]. Hence, personality traits, particularly neuroticism, may also be an important factor influencing CCs and presenteeism in adult workers. However, personality traits were not evaluated in the present study, which could be a limitation. The mediating role of personality traits on the relationship among parental overprotection and CCs or presenteeism should be investigated in future studies. In adult workers, parental overprotection (i.e. POP and MOP) decreases resilience and increases work stress [27]. In addition, work stress directly exacerbates presenteeism in office workers [28]. Hence, parental overprotection may affect presenteeism via resilience or work stress. However, resilience and work stress were not evaluated in this study, which could also be a limitation. The mediating role of resilience and work stress on the relationship among parental overprotection and presenteeism should also be investigated in future studies.

This study suggests that PCA and MCA decrease CCs and presenteeism. Parental care (i.e. PCA and MCA) increases resilience and decreases stress response of adult workers [27]. In addition, psychological and physical stress response directly exacerbates presenteeism [28]. Hence, in this study, the protective role of PCA and MCA on presenteeism is consistent with evidence obtained from a previous study [27]. In the present study, there was a gap between PCA and MCA in the mediating role of CCs on presenteeism. More specifically, the influence of PCA and MCA on presenteeism were fully and partially mediated by CCs, respectively. A previous study reported that PCA as well as MCA decreased neuroticism [6], which could lead to decreased presenteeism. Furthermore, the quality of childhood parenting care by same-sex parents affects interpersonal sensitivity in Japanese adults [29]. Hence, the role of PCA and MCA on presenteeism could vary by sex in Japanese workers, which should be investigated in future studies. In the previous study, MCA, but not PCA, significantly and negatively predicted the occurrence of DSs in general adults [19]. DSs directly affect presenteeism [10]; hence, MCA may have reduced presenteeism by decreasing DSs. However, DSs were not evaluated in this study, which could be another limitation. Therefore, the mediating role of DSs on the relationship among parental care (PCA and MCA) and presenteeism should be investigated in the future.

The role of CCs in workers may gain more importance in the future, because cognitive symptoms contribute the most to presenteeism [30]. In addition, quality of life is more closely associated with CCs than objective cognitive dysfunction evaluated by neurocognitive assessments [18]. In terms of clinical importance, CCs mediate the impact of temperament characteristics on disability [20]. In workers, CCs mediate the impact of DSs on presenteeism [10]. Childhood parental bonding impacts workers’ MH by influencing their stress-coping ability and reactions to stress [9]. Thus, the mediating role of CCs on the relationship among childhood parental bonding and presenteeism may contribute to the variations in the stress-coping ability and reactions to stress of workers. Recently, personality characteristics have been identified as important factors for the effectiveness of interventions in MH [31]. Therefore, to better understand the role of CCs in occupational MH and develop interventions against presenteeism, the mediating role of CCs on the relationship between personality characteristics and presenteeism should be investigated in future studies.

This study suggests that there may be a difference between general adult workers and non-psychiatric workers in the role of CCs in the relationship between childhood parental bonding and presenteeism. Accurate assessment of cognitive dysfunction may be difficult in individuals with psychiatric illness [14, 15], which could have affected our findings. Furthermore, in Japanese adults, childhood parental bonding affects affective temperaments, and affective temperaments mediate the influence of childhood parental bonding on CCs [19]. Affective temperaments also influence occupational stress in Japanese workers [32, 33]. Therefore, affective temperaments could be confounding factors in the relationship between childhood parental bonding, CCs, and presenteeism, which should be investigated in future studies.

Regarding the limitations of this study, the cross-sectional design could not determine the causal linkages among the parameters. This research study was conducted in Japan, which may prevent generalization of the results to other communities. The participants of this research were adults, which could prevent generalization of our findings to adolescents or children. Furthermore, this study recruited only currently employed individuals, which could prevent generalization of our findings to unemployed individuals. Both healthy and unhealthy workers were concurrently recruited and analyzed in the present study. Objective assessments of participants with current psychiatric treatment and psychiatric history were not conducted, which could be a limitation [34, 35]. Finally, the influence of memory bias could not be corrected, because self-administered scales were utilized in this research.

## Conclusion

This study suggested that CCs mediate the influence of childhood parental bonding on presenteeism in Japanese adult workers. Evaluation of the mediating effect of CCs may be useful in occupational MH to address the presenteeism associated with childhood parental bonding. Furthermore, the evaluation of the role of CCs may attract more research attention in the future for the development of interventions against presenteeism.

## Supporting information

S1 Checklist STROBE statement—checklist of items that should be included in reports of observational studies. (DOCX)
